# Improvement of the Enzyme Performance of Trypsin via Adsorption in Mesoporous Silica SBA-15: Hydrolysis of BAPNA

**DOI:** 10.3390/molecules18011138

**Published:** 2013-01-16

**Authors:** Shanshan Li, Zhuofu Wu, Ming Lu, Zhi Wang, Zhengqiang Li

**Affiliations:** 1 Key Laboratory for Molecular Enzymology and Engineering of the Ministry of Education, College of Life Sciences, Jilin University, Changchun 130012, China; 2 College of Life Sciences and Agriculture and Forestry, Qiqihar University, Qiqihar 161006, China; 3 State Key Laboratory of Inorganic Synthesis and Preparative Chemistry, College of Chemistry, Jilin University, Changchun 130012, China

**Keywords:** SBA-15 mesoporous silica, trypsin, immobilization, specific activity, hydrolysis, BAPNA

## Abstract

The enzymatic performance of trypsin in hydrolysis of *N*-α-benzoyl-DL-arginine-4-nitroanilide (BAPNA) was improved by adsorption on Santa Barbara Amorphous (SBA)-15 mesoporous silica. The optimal immobilization conditions were screened and the properties of immobilized enzyme have also been studied. Under the optimal conditions, the immobilized trypsin displays maximum specific activity (49.8 μmol/min/g). The results also indicate that the immobilized trypsin exhibits better storage stability.

## 1. Introduction

The technique of enzyme immobilization plays an important role in modern biotechnology [1]. It can not only stabilize the quaternary structure of enzymes [2,3], but also maintain or enhance enzyme activity in comparison to the free enzyme [4,5].

During the last decades, the mesoporous silica has become the perfect carrier due to its tailorable pore size in the range 2~30 nm, high special surface area and strong adsorption capability [6]. The enzymes immobilized on mesoporous silica shows perfect stabilization and excellent reusability in catalytic reactions as compared to free enzymes. After grafting with the acid, basic, hydrophilic or hydrophobic group, functionalized mesoporous molecule sieves yielded new characteristics which enhanced the affinity between the silica matrix and the enzyme and induced an increase of the specific activity [7]. So far, mesoporous molecule sieves have already been used to immobilize many enzymes [8,9]. Undoubtedly, physical adsorption was one of the simplest, the mildest and the most effective methods of enzyme immobilization, compared with other methods such as encapsulation, or covalent attachment [10] and many reports have focused on enzyme adsorption on ordered mesoporous silica [11,12,13].

SBA-15 mesoporous silica possesses thick uniform silica walls (3.1 to 6.4 nm) as compared to the other mesoporous silicas such as MCM-41 or MCM-48, which provides SBA-15 with better stability. Hence, many researchers have studied the applications of this material. In 2008, the adsorption of penicillin acylase was performed using amino-functionalized mesoporous silica SBA-15 [14]. The immobilized PGA enzyme retained its activity without significant loss after the ten cycles. Yin *et al*. investigated the adsorption of lysozyme on mesoporous SBA-15 rods with tunable pore length [15]. Monduzzi and coworker synthesized mesoporous silica SBA-15 and used it as a support for the immobilization of lipase by physical and chemical adsorption [16]. The lysozyme adsorption kinetics on SBA-15 could be described according to a pseudo-second order model or an intraparticle diffusion model [17]. Owing to Kim and colleges’ report, the pore expander SBA-15 was used to adsorb the bovine carbonic anhydrase for CO_2_ sequestration [18]. Cheng *et al*. demonstrated that the short-channel SBA-15-p materials provided higher adsorption capacities and adsorption rates with cytochrome c compared to conventional fiber-like SBA-15 materials [19]. According to the report of Tan and colleagues, lipase was adsorbed into aminopropyl-grafted mesoporous silica nanotubes to achieve the resolution of (*R,S*)-1-phenylethanol [20]. Immobilized lipase on aminopropyl-grafted mesoporous silica nanotubes acquired higher activity than immobilized lipase on mesoporous silica nanotubes owing to the reformation of secondary structure of lipase after the immobilization. In Tumbiolo’s report, pepsin was immobilized in mesoporous silica SBA-15 by physical adsorption. The pore openings of the mesoporous silica SBA-15 was reduced by grafting organosilane groups to minimize the leaching of pepsin [21]. It can be concluded that SBA-15 is a perfect carrier for enzyme immobilization.

However, poor results were obtained when SBA-15 was used as carrier to immobilize trypsin [22]. The main problem is the rapid autolysis of trypsin, which may severely decrease the performance of the immobilization trypsin. Addition of some stabilizing agents can overcome this problem. Michael has reported that use of a CD-containing polymer as an additive could improve the functional stability of trypsin in aqueous solutions [23]. They found that trypsin was 6-fold more resistant to autolytic inactivation in the presence of the additive. Moreover, trypsin was chemically modified by derivatives of cyclodextrin, and the increased resistance of CD-trypsin conjugates to autolysis and temperature was observed [24,25]. In this manuscript, the trypsin was adsorbed into SBA-15 with β-cyclodextrin as stabilizing agents. Immobilization conditions were screened and the properties of immobilized enzyme have also been evaluated.

## 2. Results and Discussion

### 2.1. Synthesis and Characteristic of SBA-15 Mesoporous Silica

**Figure 1:**
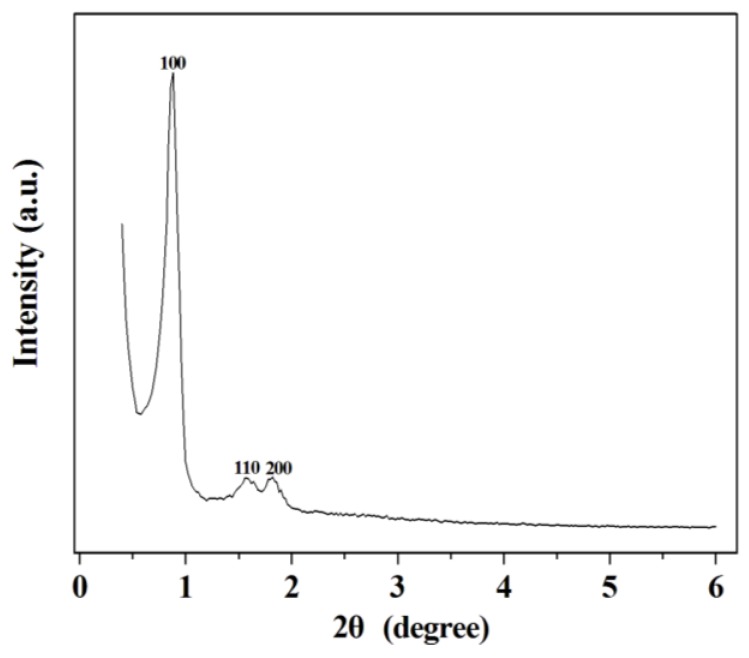
Powder XRD pattern of mesoporous silica SBA-15.

**Figure 2:**
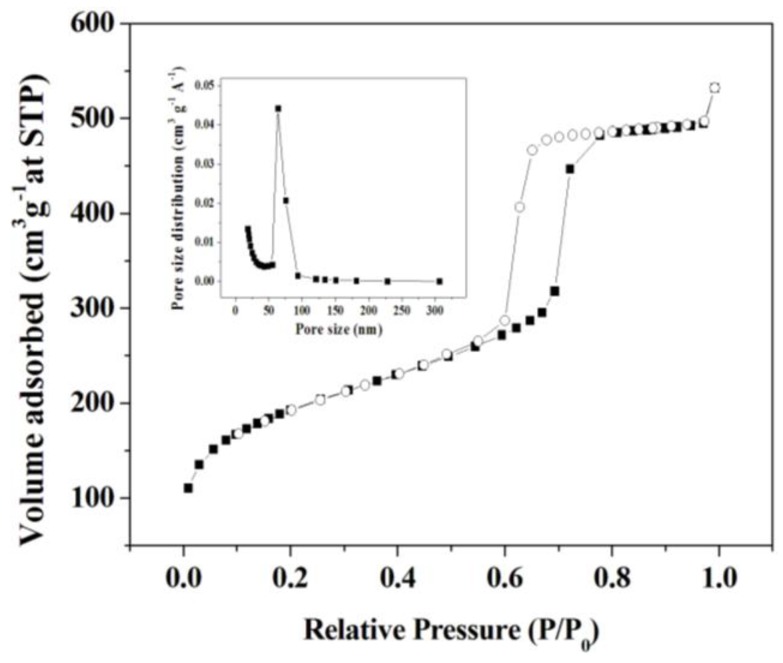
Nitrogen adsorption-desorption isotherms and BJH pore size distributions of mesoporous silica SBA-15: adsorption curve (■) and desorption curve (○).

The small-angle X-ray pattern shows three well-resolved peaks indexed to (100), (110), and (200) reflections corresponding to a two-dimensional hexagonal *P*6*mm* structure with a large unit-cell parameter (Figure 1) [26]. The nitrogen adsorption-desorption isotherm at 77 K (Figure 2) can be classified as type IV and exhibits an H_1_-type hysteresis at high relative pressure [27]. These were typical features of mesoporous silica SBA-15. H_1_ hysteresis reveals the presence of open channels which facilitates the effective diffusion of substrates and product. It is well known that the size matching between enzyme molecular and host matrix pore plays a key role in achieving high enzyme loadings [11]. As shown in the inset of Figure 2, the narrow Barrett-Joyner-Halenda (BJH) distribution of the synthesized SBA-15 is 63 Å, which is appropriate for the adsorption of trypsin (it’s spherical diameter is about 3.8 nm) [28].

### 2.2. Influence of Immobilization Conditions on Trypsin Activity

#### 2.2.1. Effect of Immobilization pH 

**Figure 3:**
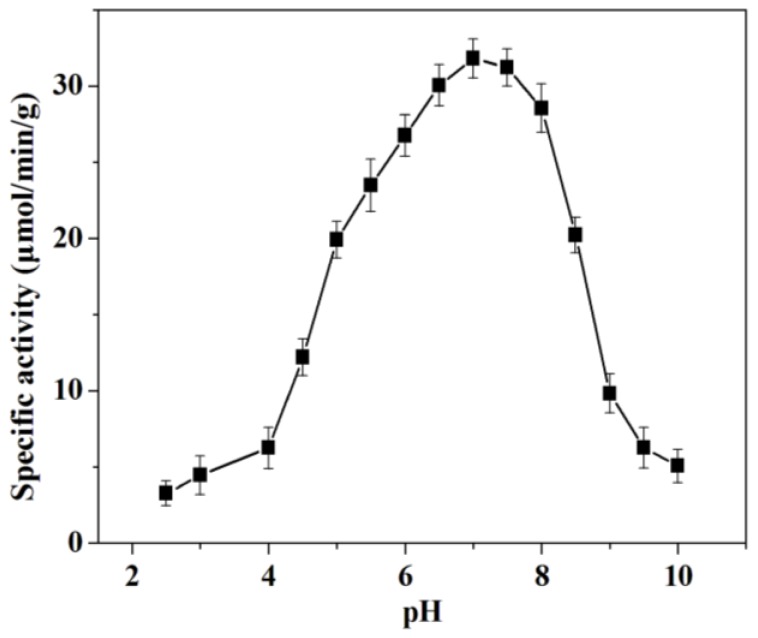
Effect of pH on the specific activity of immobilized trypsin.

The charge state of protein and mesoporous silica SBA-15 can be modulated by changing pH value of the solution, which can alter the electrostatic forces between mesoporous silica SBA-15 and trypsin. To clarify the influence of pH of the immobilization solution on immobilization process, the specific activity of the immobilized trypsin prepared was detected in different pH solutions. As shown in Figure 3, the specific activity gradually increases in the pH range of 2.5~7.0, and decreased sharply from pH 7.0 to 10.0. The maximum specific activity was observed at pH 7.0. It’s known that the isoelectric points of mesoporous silica SBA-15 and the trypsin are 2.1 [6] and 10.5 [11], respectively. When the immobilization process was carried out in the solution with the pH in the range of 2.5~10.0, the opposite charges of mesoporous silica SBA-15 and trypsin were beneficial for the adsorption. However, the denaturation of enzyme caused by low pH (2.5~6.0) or high pH (8.0~10.0) decreases the specific activity of immobilized trypsin.

#### 2.2.2. Effect of the Ratio of Trypsin to β-Cyclodextrin

**Figure 4:**
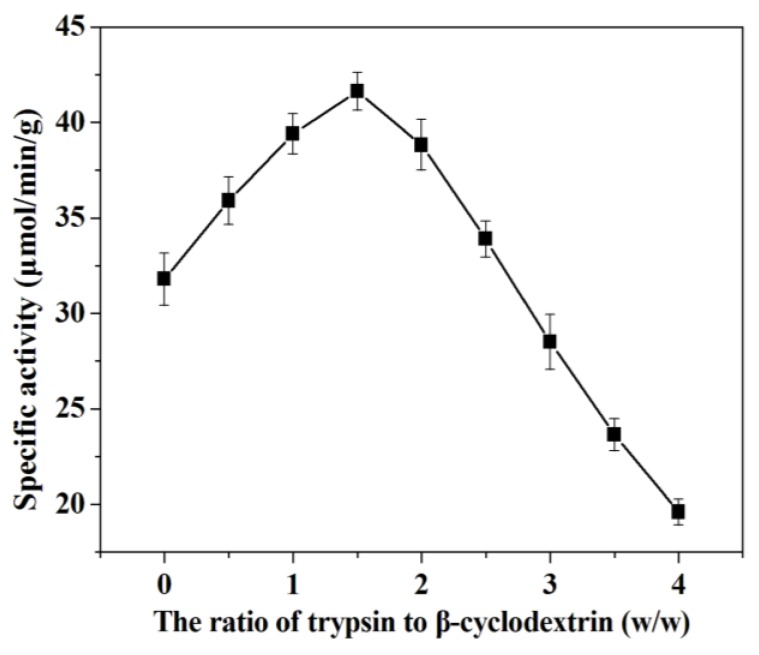
Effect of the ratio of trypsin to β-cyclodextrin on the specific activity of immobilized trypsin.

It’s well known that the lyophilization during immobilization involves inherent destabilization forces that can denature the enzyme e.g., cold shock, ice-water interfaces, dehydration stress, *etc*. [29], so in this study, β-cyclodextrin was selected as lyoprotectant to protect trypsin during immobilization. The effect of the ratio of trypsin to β-cyclodextrin has been investigated in the range from 1:0.5 to 1:4 (w/w) and the results are shown in Figure 4. The maximum specific activity was obtained at the ratio 1:1.5 (w/w). The effect of additives on the activity of immobilized trypsin is not yet well understood. It’s generally believed that the presence of β-cyclodextrin during the immobilization process can give protection to trypsin against the damage from lyophilization [30,31]. Furthermore, β-cyclodextrin might decrease the autolysis of the trypsin during the immobilization process [23]. However, β-cyclodextrin at high concentrations could destroy the electrostatic attraction between trypsin and mesoporous silica SBA-15, which led to the decrease of adsorbed amount.

#### 2.2.3. Effect of Immobilization Time 

**Figure 5:**
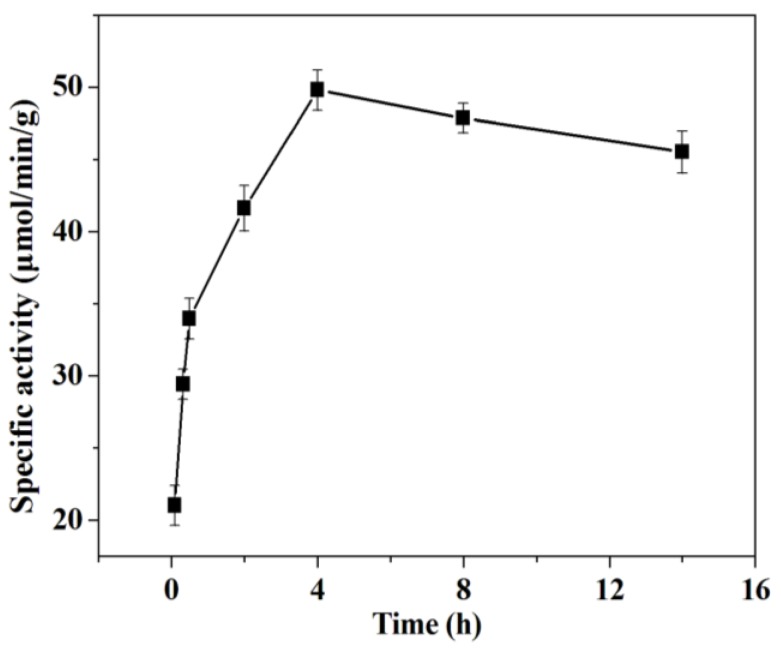
Effect of immobilization time on the specific activity of immobilized trypsin.

The effect of immobilization time on the enzyme activity was studied in the range of 0.1~22 h. It can be observed from Figure 5 that the enzyme activity exhibits a bell shaped curve with changing immobilization time and the maximum enzyme activity was observed at 4 h. In this study, the initial trypsin amount used was beyond the adsorption capability of mesoporous silica SBA-15. The specific activity of immobilized trypsin increases with the extension of immobilization time at the initial stage (0–4 h), which might mainly be due to the increase in trypsin loading amount. The specific activity of trypsin approaches the maximum value at an immobilization time of 4 h, suggesting that the monolayer adsorption of trypsin reached its saturation point. When the immobilization time was too long, the over-loading of trypsin could lead to a multi-layered stacking of enzyme, which would block the substrate molecules from accessing to the internal trypsins and thus decreased the enzyme activity. Such a result is consistent with a published report in which the multilayer adsorption of lipase caused the decreased of enzyme activity [32]. Moreover, the self-hydrolysis of trypsin became more and more serious with the increase of immobilization time. This result is in accordance with Lei’s report in which the specific activity of entrapped glucose oxidase in mesoporous silica decreased with increasing protein loading density [33].

### 2.3. Enzyme Performance of the Immobilized Trypsin

#### 2.3.1. Reusability

**Figure 6:**
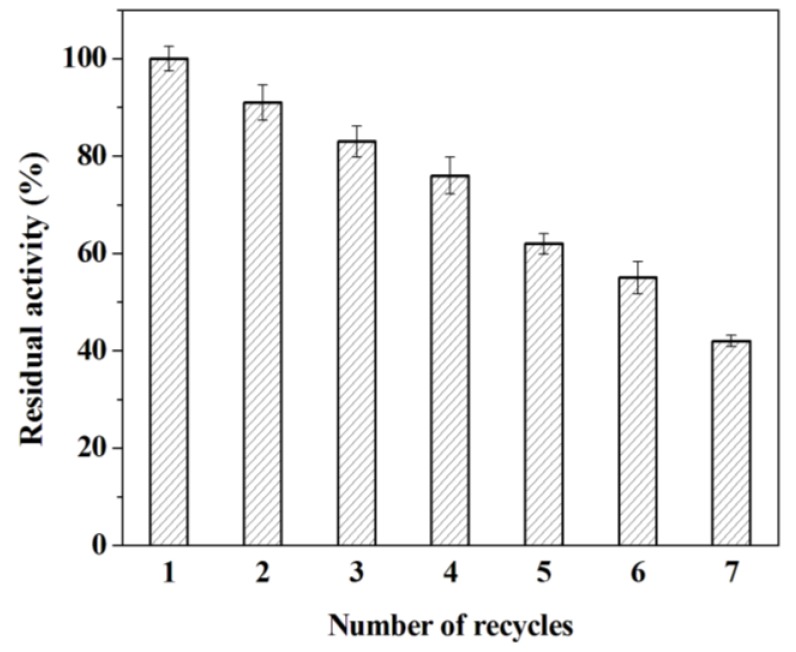
Catalyst recycling of the immobilized trypsin. The specific activity of immobilized trypsin (49.8 μmol min^−1g−1^) in the first cycle was taken as control (100%).

Generally speaking, the enzymes dissolved in reaction medium are often difficult to recover, let alone reuse. Therefore, the reusability of immobilized enzyme deserves further research. To check this parameter, the immobilized trypsin was used in subsequent cycles for the hydrolysis of BAPNA. As seen in Figure 6, the enzyme activity of adsorbed trypsin decreases slightly as the number of recycles increases. After seven cycles, the adsorbed trypsin still retains 42% of initial activity. The lost of enzyme activity shall be mainly attributed to the leaching of adsorbed trypsin at the repeated reaction, separation and rinsing steps in successive reaction owing to the weak electrostatic interaction between trypsin and mesoporous silica SBA-15. Furthermore, the leached trypsin increases the possibility of the autolysis.

#### 2.3.2. Storage Stability

**Figure 7:**
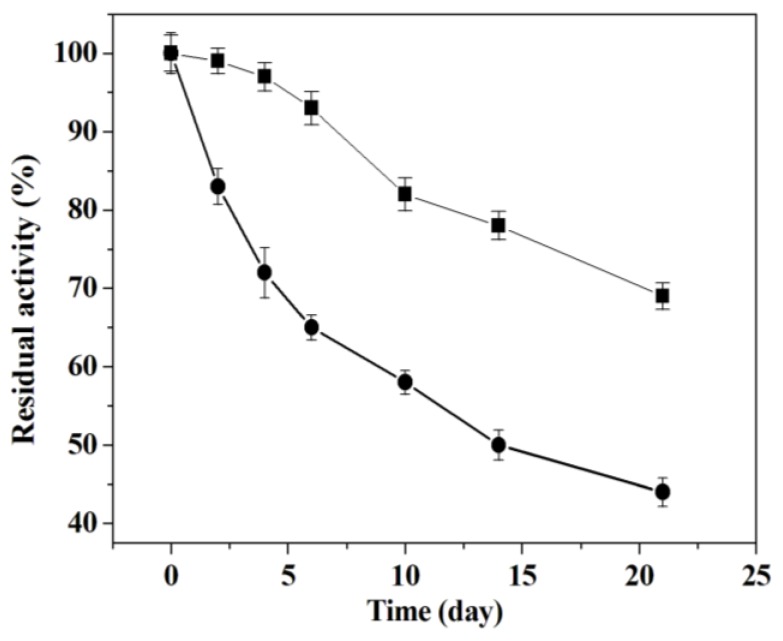
Storage stability of the immobilized (■) and free trypsin (●). For free trypsin, the specific activity (0.46 μmol min^−1^ mg^−1^) at 0 h was taken to be 100%. For immobilized trypsin, the specific activity (49.8 μmol min^−1 g−1^) at 0 h was taken to be 100%.

The storage stability of immobilized and free enzyme was investigated at 4 °C for 3 weeks. Figure 7 presents that the adsorbed trypsin conserved 64% of initial activity, whereas the free trypsin maintained 44% of initial activity in 21 days.

This enhanced stability was due to the inhibition of autolysis after adsorption. After immobilization, the positively charged trypsin can be tightly attached to the negatively charged silica wall, and then the collision between entrapped trypsins is restricted, which lowered the autolysis of the trypsin. However, unlike the anchoring bonds of covalently attached enzymes, lowered electrostatic interaction between adsorbed trypsin and mesoporous silica SBA-15 leads to the leakage of the trypsin and weakens the storage stability. Bein and coworker had immobilized trypsin in the channel of large-pore SBA-15 by a click chemistry approach and verified that no trypsin leaching was found after immobilization [34]. In Jasra’s work, alkaline serine endopeptidase was immobilized on surface-modified SBA-15 through amide bond formation by using carbodiimide as a coupling agent and it was demonstrated that immobilized enzymes showed higher specific activity than the free enzyme, which was ascribed to easy accessibility of substrate molecules to active sites of immobilized enzyme [35]. In order to improve the reusability and storage stability of immobilized trypsin, the trypsin immobilization by covalent attachment method is being worked on and will be published in due course.

## 3. Experimental

### 3.1. Materials

TEOS and Pluronic P123 (EO20-PO70-EO20, MW = 5800) were commercially available from Sigma-Aldrich (St. Louis, MO, USA). Trypsin from the bovine pancreas (EC 3.4.21.4) and BAPNA were also obtained from Sigma-Aldrich. β-Cyclodextrin and all other reagents were of analytical grade. All aqueous solutions were prepared with Milli-Q water.

### 3.2. The Synthesis of Mesoporous Molecular Sieve

P123 (0.8 g) was dissolved in distilled water (25 mL). Then HCl (12 M, 3 mL) and TEOS (2.4 g) were added. The system was stirred for 24 h at 40 °C, followed by crystallization for 48 h at 100 °C in the reaction kettle. After calcination at 550 °C for 5 h, the template in mesoporous silica SBA-15 was removed. The mesoporous structure of mesoporous silica SBA-15 was characterized by X-ray powder diffraction (XRD, Siemens, D5005, Karlsruhe, Germany) pattern using nitrogen as balance air and hydrogen as carrier air. The specific surface area was assessed through the Brunauer-Emmett-Teller (BET) method, and the pore size distribution curve was computed using the Barrett-Joyner-Halenda (BJH) method (ASAP 2010M, Micromeritics Inc., Norcross, GA, USA).

### 3.3. Preparation the Immobilization Solution

Trypsin (1 g) was dissolved in K_2_HPO_4_-KH_2_PO_4_ buffer (100 mL, pH 7.0, 0.05 M,) and the insoluble impurity was removed by centrifugation (8,000 rpm, 5 min) at 4 °C. Finally the supernatant was lyophilized. The immobilization solution [10 mg/mL of trypsin, containing β-cyclodextrin (15 mg/mL)] was prepared by redissolving the lyophilized trypsin in the K_2_HPO_4_-KH_2_PO_4_ (pH 7.0, 0.05 M) buffer at 4 °C.

### 3.4. The Preparation of Adsorbed Trypsin

The aliquot of mesoporous silica SBA-15 solid powders (1 g) was immersed in immobilization solution (100 mL) for 4 h with stirring at 4 °C. After centrifugation at 4,000 rpm for 7 min, the resulting product was repeatedly washed by the buffer with the same pH, until the UV absorbance (280 nm) in the supernatant disappeared. Finally, the resultant sample was lyophilized, and then stored at −20 °C. The final product was white powder.

### 3.5. Optimization of the Immobilization Process

The whole procedure of adsorption was implemented at 4 °C to reduce the inactivation and autolysis of trypsin.

#### 3.5.1. Optimization of Immobilization pH 

The buffers with different pH value (2.5~10.0) were used to optimize the pH during immobilization process at 4 °C. The used buffers were citric acid-sodium citrate (pH 2.5–4.6), K_2_HPO_4_-KH_2_PO_4_ (pH 5.8–8.0) and Gly-NaOH (pH 9.3–10.0), respectively. The specific activity of the immobilized trypsin was assayed according to the description in Section 3.6.

#### 3.5.2. Optimization of the Ratio of Trypsin to β-Cyclodextrin

The adsorption process of trypsin was carried out in K_2_HPO_4_-KH_2_PO_4_ buffer at 4 °C (pH 7.0, containing different amount of β-cyclodextrin). After lyophilization, the specific activity of the immobilized trypsin was measured following the procedure in Section 3.6.

#### 3.5.3. Optimization of Immobilization Time

The mesoporous silica SBA-15 solid powders (1 g) were immersed into trypsin solution (100 mL) for different immobilization times (0.1~22 h) at 4 °C. Then the specific activity of the immobilized trypsin was measured following the procedure in Section 3.6.

### 3.6. Enzyme Activity Assay

The enzyme activity of trypsin was measured using BAPNA as substrate. The reaction system contained BAPNA (100 µL, 10 mM) and K_2_HPO_4_-KH_2_PO_4_ buffer (800 µL, 0.05 M, pH 7.0). The hydrolytic reaction was triggered by adding the enzyme (5 mg). Then, the reaction mixture was incubated in water bath at 25 °C for 5 min with magnetic stirring. Thereafter, the reaction was stopped by adding 30% acetic acid solution (0.5 mL) followed by centrifuging at 12,000 rpm for 3 min. The amount of *p*-nitroaniline produced was determined at 410 nm. The specific activity (μmol/g/min) was defined as the amount (in micromoles) of *p*-nitroaniline produced per minute per gram of SBA-15 loaded with the trypsin.

### 3.7. Reusability

The immobilized trypsin was used for successive batches. The relative activity of adsorbed trypsin was measured according to the assay described in Section 3.6. After each cycle, the reaction mixture was centrifuged at 12,000 rpm for 3 min. The obtained precipitation was washed with K_2_HPO_4_-KH_2_PO_4_ buffer (pH 7.0, 0.05 M) to remove any residual substrate or product and then kept overnight in a vacuum oven for a complete drying. After the centrifugation, washing and drying, the powder was used at next cycle under otherwise equivalent conditions. The residual activity of the recycled enzyme was compared with the enzyme activity of the first cycle (100%).

### 3.8. Assessment of Storage Stability

The immobilized trypsin was kept in K_2_HPO_4_-KH_2_PO_4_ buffer (pH 7.0, 0.05 M) at 4 °C for 21 days. With BAPNA as substrate, the residual activity was checked from time to time according to the description in Section 3.6. The relative activity of immobilized trypsin at specific storage time was compared with the enzyme activity of immobilized trypsin before storage (100%). For free trypsin, the storage stability was measured according to above mentioned process.

### 3.9. Statistical Analysis

The data expressed in various studies was plotted using Sigma Plot-9 and expressed as standard error (±). Each value represents the mean for three independent experiments performed in duplicate, with average standard deviation <5%.

## 4. Conclusions

In this manuscript, trypsin was immobilized in mesoporous silica SBA-15 by physical adsorption to obtain optimal specific activity for the hydrolysis of BAPNA. When the immobilization of trypsin had been performed in pH 7.0 buffers for 4 h and the ratio of trypsin to β-cyclodextrin was 1.5 in the adsorption system, the optimal enzyme activity was obtained. Under the optimum conditions, the native conformation of trypsin is retained and the overlay or aggregation between the immobilized enzyme molecules is maximally avoided. Most importantly, β-cyclodextrin provided protection for trypsin during the immobilization process. After seven recycles, immobilized trypsin maintains 42% of initial activity, and the loss of enzyme activity is mainly due to the leakage of the enzyme. During 30 days, the immobilized trypsin exhibits better storage stability than the free one, which is attributed to the protection from the hydroxyls of β-cyclodextrin and SBA-15 mesoporous silica. On the basis of this work, the protective effect of additive (polyol) on enzyme immobilization and stabilization in mesoporous silica material will be investigated, particularly in non-conventional media. In future work, the resultant immobilized trypsin will be used to synthesize amino acid esters in organic media.
